# Dimensional reduction based on peak fitting of Raman micro spectroscopy data improves detection of prostate cancer in tissue specimens

**DOI:** 10.1117/1.JBO.26.11.116501

**Published:** 2021-11-06

**Authors:** Arthur Plante, Frédérick Dallaire, Andrée-Anne Grosset, Tien Nguyen, Mirela Birlea, Jahg Wong, François Daoust, Noémi Roy, André Kougioumoutzakis, Feryel Azzi, Kelly Aubertin, Samuel Kadoury, Mathieu Latour, Roula Albadine, Susan Prendeville, Paul Boutros, Michael Fraser, Rob G. Bristow, Theodorus van der Kwast, Michèle Orain, Hervé Brisson, Nazim Benzerdjeb, Hélène Hovington, Alain Bergeron, Yves Fradet, Bernard Têtu, Fred Saad, Dominique Trudel, Frédéric Leblond

**Affiliations:** a Centre de recherche du Centre hospitalier de l’Université de Montréal, Montreal, Quebec, Canada; b Institut du cancer de Montréal, Montreal, Quebec, Canada; c Polytechnique Montréal, Department of Engineering Physics, Montreal, Quebec, Canada; d Université de Montréal, Department of Pathology and Cellular Biology, Montreal, Quebec, Canada; e Polytechnique Montréal, Department of Computer Engineering and Software Engineering, Montreal, Quebec, Canada; f Centre hospitalier de l’Université de Montréal, Department of Pathology, Montreal, Quebec, Canada; g University Health Network, Laboratory Medicine Program, Toronto, Ontario, Canada; h Informatics & Biocomputing Program, Ontario Institute for Cancer Research, Toronto, Ontario, Canada; i University of California, Los Angeles, Department of Human Genetics, Los Angeles, California, United States; j University of California, Los Angeles, Department of Urology, Los Angeles, California, United States; k University of California, Los Angeles, Institute for Precision Health, Los Angeles, California, United States; l University of California, Los Angeles, Jonsson Comprehensive Cancer Center, Los Angeles, California, United States; m University Health Network, Princess Margaret Cancer Centre, Toronto, Ontario, Canada; n Centre de recherche du Centre hospitalier universitaire de Québec-Université Laval, Oncology Division, Quebec City, Quebec, Canada; o Université Laval, Centre de recherche sur le cancer, Quebec City, Quebec, Canada; p Université Laval, Department of Surgery, Quebec City, Quebec, Canada

**Keywords:** machine learning, Raman micro-spectroscopy, prostate cancer, feature selection, feature reduction

## Abstract

**Significance:** Prostate cancer is the most common cancer among men. An accurate diagnosis of its severity at detection plays a major role in improving their survival. Recently, machine learning models using biomarkers identified from Raman micro-spectroscopy discriminated intraductal carcinoma of the prostate (IDC-P) from cancer tissue with a ≥85% detection accuracy and differentiated high-grade prostatic intraepithelial neoplasia (HGPIN) from IDC-P with a ≥97.8% accuracy.

**Aim:** To improve the classification performance of machine learning models identifying different types of prostate cancer tissue using a new dimensional reduction technique.

**Approach:** A radial basis function (RBF) kernel support vector machine (SVM) model was trained on Raman spectra of prostate tissue from a 272-patient cohort (Centre hospitalier de l’Université de Montréal, CHUM) and tested on two independent cohorts of 76 patients [University Health Network (UHN)] and 135 patients (Centre hospitalier universitaire de Québec-Université Laval, CHUQc-UL). Two types of engineered features were used. Individual intensity features, i.e., Raman signal intensity measured at particular wavelengths and novel Raman spectra fitted peak features consisting of peak heights and widths.

**Results:** Combining engineered features improved classification performance for the three aforementioned classification tasks. The improvements for IDC-P/cancer classification for the UHN and CHUQc-UL testing sets in accuracy, sensitivity, specificity, and area under the curve (AUC) are (numbers in parenthesis are associated with the CHUQc-UL testing set): +4% (+8%), +7% (+9%), +2% (6%), +9 (+9) with respect to the current best models. Discrimination between HGPIN and IDC-P was also improved in both testing cohorts: +2.2% (+1.7%), +4.5% (+3.6%), +0% (+0%), +2.3 (+0). While no global improvements were obtained for the normal versus cancer classification task [+0% (−2%), +0% (−3%), +2% (−2%), +4 (+3)], the AUC was improved in both testing sets.

**Conclusions:** Combining individual intensity features and novel Raman fitted peak features, improved the classification performance on two independent and multicenter testing sets in comparison to using only individual intensity features.

## 1 Introduction

Intraductal carcinoma of the prostate (IDC-P) is an aggressive variant of prostate cancer (PC) recognized as a distinct entity in 2016 by the World Health Organization classification.^1^ Current biomarkers used by pathologists for IDC-P identification, phosphatase and tensin homolog loss, and ETS-related gene overexpression, have low sensitivity limiting their use. However, by comparing PC and IDC-P Raman spectra, high sensitivity (≥85%) biomarkers were recently identified in our previous work and led to the first machine learning model for the diagnosis of IDC-P using Raman micro-spectroscopy (RμS).^2^ In this past study, Raman spectra from three institutes were collected independently to distinguish various types of prostate cancer on completely independent testing sets. Employing Raman spectroscopy for the identification of various pathologies is currently well-established.^3^

One of the main challenges of using machine learning algorithms on Raman spectra is to create, extract, and select features. Most common techniques consist of using individual intensities and various complex feature selection methods, such as recursive feature elimination,^4^ ant colony optimization,^5^^,^^6^ and $L_{0}$-SVM, or adaptive boosting,^7^ to rank them and select the most relevant ones. While some of these methods are much more powerful than others when only a few dozens of individual Raman intensities are considered, all methods provide a very similar classification accuracy when the number of retained features is higher than 50.^8^ Linear discriminant analysis accompanied with principal component analysis (PCA) is the most common dimensionality reduction technique used in Raman spectroscopy.^9^^–^^14^ However, our group showed that Raman peak fitting features have better predictive performances than PCA for cancer/benign brain tissue classification.^15^

In our previous study, features consisted of individual intensities of Raman bands, and the machine learning algorithm was a linear support vector machine (L1-SVM) for feature selection and an SVM with a radial basis function kernel (RBF-SVM) for classification.

In this new analysis, the same dataset, features, and machine learning architecture were kept, but a new set of features was added. Those features were obtained by fitting a Gaussian function on Raman peaks and extracting their heights and widths. Individual intensities of Raman bands can be any band within a Raman peak, whereas fitted heights capture only the maximum of peaks, but they differ since they are much less prone to stochastic noise. The algorithm used to extract these new features is an improved version of the Raman peak fitting algorithm used in Ref. 15. This algorithm was initially designed to extract height and width features of Raman peaks to improve the interpretability of Raman signal in brain tissue by fitting only Raman peaks that are constantly present in the brain Raman literature.

This present study aims to demonstrate that these two sets of features are complementary and thus improve the classifying results of three PC tissue classification tasks: benign versus cancer, cancer versus IDC-P, and high-grade prostatic intraepithelial neoplasia (HGPIN) versus IDC-P, where the cancer dataset does not contain any IDC-P spectra and the benign dataset does not contain any HGPIN spectra. Major modifications to the algorithm consisted of implementing an algorithm that the finds most common peaks without requiring a previously established list of Raman peaks, and improving the algorithm that identifies inflection points of peaks to obtain more accurate width of peaks especially in signal regions with stochastic noise.

## 2 Methodology

### 2.1 Patient Samples and Imaging Method

The dataset consisted of 483 PC patients from three different institutions: Centre hospitalier de l’Université de Montréal (CHUM), University Health Network (UHN), and Centre hospitalier universitaire de Québec-Université Laval (CHUQc-UL). The characteristics of the dataset are shown in Table 1. Tumor stages defines where cancer is present in prostate tissue; there are three tumors stages: pT2 (organ confined), pT3a (extraprostatic extension or microscopic invasion of bladder neck), and pT3b (seminal vesicle muscle wall invasion). The Gleason score details the arrangement and pattern of cancer cells. There are five patterns, and the Gleason score is equal to the sum of the most and second most common pattern present prostate tissues. Confocal RμS measurements on formalin-fixed paraffin embedded tissue microarrays (TMAs) of PC and IDC-P tissue were acquired as described in our previous work.^2^ Briefly, sections from the TMAs were transferred onto aluminum slides with low Raman activity (Miro5011, Anomet, Brampton, Ontario) and dewaxed according to the CHUM standard clinical dewaxing protocol. Raman acquisitions were made using a confocal Raman microscope (Renishaw, Gloucestershire) equipped with a 785-nm line focus laser and a grating of 1200 lines/mm allowing acquisition of Raman shifts between 602 and $1726\;{\mathrm{cm}}^{-1}$. For each TMA, five accumulations of 10 s each at a 150-mW laser output power were acquired using a 50× objective with a 0.75 numerical aperture. There were always four spectra taken per TMA core at four different locations, whereas the number of TMA cores per patient varied between institutions. The diameter of TMA cores ranged between 0.6 to 1.2 mm and the probed region corresponded to a rectangular area of $24\;\mu m^{2}$^2^ (8  μm×3  μm, approximately corresponding to single-cell analysis). IDC-P identification was done by two independent pathologists. All Raman spectra files are available in the Dryad Digital Repository database.^16^

**Table 1:** Patient clinicopathological characteristics separated by institution: CHUM, Centre hospitalier de l’Université de Montréal; CHUQc-UL, Centre hospitalier universitaire de Québec–Université Laval; IDC-P, intraductal carcinoma of the prostate; IQR, interquartile range; PSA, prostate-specific antigen; TMA, tissue microarray; UHN, University Health Network.

Characteristic	Institution		
	Training	Testing	
	CHUM	UHN	CHUQc-UL
Number of patients	272	76	135
Median age in years at radical prostatectomy (IQR)	62 (58-66)	61 (57-66)	62 (59-67)
Median pre-operative PSA in μg/l (IQR)	7.4 (5.1-11.9)	6.9 (5.2-10.7)	6.6 (4.9-9.1)
Radical prostatectomy gleason score, n (%)	265	67	133
≤3+3	139 (52)	14 (21)	10 (8)
3+4	74 (28)	34 (51)	69 (52)
4+3	22 (8)	14 (21)	42 (32)
≥4+4	30 (11)	5 (7)	12 (9)
Pathological tumor stage, n (%)	270	72	134
pT2	185 (69)	32 (44)	77 (57)
pT3a	60 (22)	32 (44)	41 (31)
pT3b	25 (9)	8 (11)	16 (12)
Presence of IDC-P among patients	15 (6)	14 (18)	15 (11)
Number of TMA cores per patient	1	1-3	1-6
Number of spectra per core	4	4	4
Number of spectra per patient	4	4-12	4-24
Number of spectra benign/cancer	99/272	49/204	68/253
Number of spectra cancer/IDC-P	272/112	191/139	253/104
Number of spectra HGPIN/IDC-P	170/112	23/22	30/28

### 2.2 Data Preprocessing

The following data preprocessing procedures are applied to obtain each spectrum: (1) summed the five accumulations of 10 s each, (2) remove cosmic rays, (3) remove background signal produced by the aluminum slides and tissue fluorescence using the rolling ball algorithm,^17^ (4) apply standard normal variate (SNV) normalization, (5) average the four spectra of each TMA core resulting in between 1 and 3 spectra per patient. During background removal, the rolling ball algorithm distorts, due to border effects, Raman signals by creating peaks that are not present in the raw spectra. To avoid these artifacts, regions between 602 and $669\;{\mathrm{cm}}^{-1}$, as well as between 1707 and $1726\;{\mathrm{cm}}^{-1}$ were removed.

A limitation of the rolling ball algorithm comes from the presence of a finite size structuring element within the algorithm. At the beginning and end of a spectrum, half of this structuring element extends past the range of spectra and causes distortions in the extracted Raman signals due to this border effect. The magnitude of this effect depends on the smoothness of the signal baseline. In this particular dataset, the baseline shows a step non-smooth gradient at the beginning of the signal, which increases the distortion, and a smooth baseline at higher wavelengths. It was determined, empirically, that to remove any residue due to this effect, signal regions between 602 and $669\;{\mathrm{cm}}^{-1}$ as well as between 1707 and $1726\;{\mathrm{cm}}^{-1}$ had to be removed, i.e., a portion of the beginning and end of each spectrum.

The next preprocessing step is to fit Gaussian functions over the most common Raman peaks present in the dataset. Since the Gaussian function is strictly positive, the minimum value of SNV normalized spectra was subtracted from the entire dataset to obtain only positive values. The peak fitting algorithm, which is an improvement of the algorithm first used in Ref. 15, considers a peak only when it appears in at least 50% of spectra at the same location or within $\pm 2\;{\mathrm{cm}}^{-1}$ and if its height is above a threshold height previously determined empirically. For every peak, both inflection points are determined as well as the height of the maximum and its position. The distance between inflection points is used to compute an approximate standard deviation (σ) and to determine the fitting domain for a nonlinear least-squares regression algorithm.^18^ This standard deviation, and the maximum and position of the peak determine the three starting points given to the regression algorithm to fit a Gaussian over the wavelength domain of a peak to obtain fitted heights and widths (standard deviation, σ). These values are then SNV normalized and used as features.

### 2.3 Statistical Analysis

**Fig. 1:**
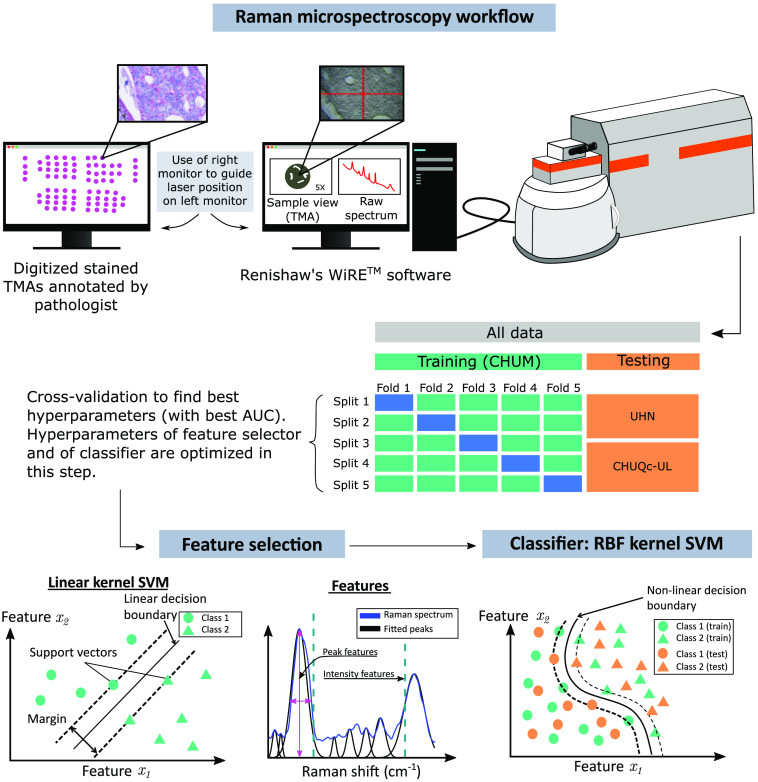
Data acquisition and machine learning workflow. Annotated stained TMAs are used to guide Raman microspectroscopy measurements. Data are split into training (CHUM) and testing sets (UHN & CHUQc-UL). A double nested five-fold CV is performed to determine the hyperparameters through a randomized grid search. Feature selections on peak and intensity features are done separately using linear kernel L1 SVM. Classification models are RBF kernel SVMs that use selected features. The hyperparameter set that yields the highest AUC on training data is used to build the final model (green), which is evaluated on predicting outcome on the testing set (orange).

A machine learning model with several hyperparameters was trained on the CHUM dataset and tested on two independent hospital cohorts: CHUQc-UL and UHN. Patient clinicopathological characteristics and number of spectra of each classification task are shown in Table 1. The model uses two types of features: individual features; intensity set, and peak features; peak set. To determine the features in each set, two separate feature selection algorithms are used. The selected features are then combined in a single feature vector and passed to the RBF kernel SVM classifier. Before training a model on the CHUM dataset, cross validation (CV) is required to determine the optimal value of the hyperparameters. For each classification task, the CV identifies the set of hyperparameters, through a grid search, that maximizes the area under the curve (AUC) of the receiver-operator-characteristic (ROC) curve of the CHUM dataset for three independent models: one model using only the intensity features, one using only the peak features, and a combined model that used both feature sets; intensity+peak. Figure 1 shows a schematic workflow of the statistical analysis.

### 2.4 Feature Engineering and Feature Selection

Two different sets of SVN normalized features were used for classification. The first set, intensity, consists of individual intensity values of single Raman bands. To extract the most relevant intensities, an L1 regularization linear SVM with one hyperparameter (regularization parameter $C_{\text{intensity}}$) is utilized for feature ranking. The linear SVM assigns coefficients to individual intensity features, which are then used to rank them by importance. Features that are close to one another, which are most likely correlated and contain the same information, are removed using the hyperparameter $n_{\text{neighbor}}$. Every feature that is $\pm n_{\text{neighbor}}\;{\mathrm{cm}}^{-1}$ from the feature with the highest coefficient is removed. The feature set is then updated, and the same procedure is applied for the feature with the second-highest coefficient and so forth. For example, if the intensity features with the highest coefficient has a Raman shift of $780\;{\mathrm{cm}}^{-1}$ and $n_{\text{neighbor}}=5$, intensity features with a Raman shift between 775 and $785\;{\mathrm{cm}}^{-1}$ will be removed. This procedure also reduces the dimensionality of the feature set.

The second set, peak, consists of Raman peaks fitted with a Gaussian distribution. The height and the width of these Gaussian fitted peaks are used as features. A linear SVM with L1 regularization (regularization parameter $C_{\text{peak}}$) is used to rank these engineered features by importance.

To further reduce the number of features within each feature set, the $k_{\text{intensity}}$ and $k_{\text{peak}}$ features with the highest ranked coefficient are used for modeling and the others are discarded. Both $k_{\text{intensity}}$ and $k_{\text{peak}}$ are hyperparameters determined during CV.

### 2.5 Modeling and Cross Validation

An RBF kernel SVM was used as a classifier. This algorithm has two hyperparameters: $C_{\mathrm{RBF}}$ and γ. A grid search was performed using CV to optimize the following hyperparameters: $C_{\mathrm{RBF}}$, $C_{\text{intensity}}$, $C_{\text{peak}}$, γ, $k_{\text{intensity}}$, $k_{\text{peak}}$, and $n_{\text{neighbor}}$. Both $k_{\text{intensity}}$ and $k_{\text{peak}}$ ranged from 5 to 100, $C_{\mathrm{RBF}}$ ranged from 0.001 to 1, γ ranged from 0.001 to 0.01, $n_{\text{neighbor}}$ from 0 to 10, and $C_{\text{intensity}}$ and $C_{\text{peak}}$ from $1\times 10^{-6}$ to $1\times 10^{-4}$. The CV scheme performed was a five repeat five-fold CV. The combination of hyperparameters generating the model with the highest AUC value was chosen to train the model using the complete training set. The selected model was then applied to the two independent testing sets.

## 3 Results

Raman spectra of the CHUM cohort were designated as the training set, due to its larger number of patients compared to the other two cohorts and to obtain a sufficiently large dataset for training purposes, and the CHUQc-UL and UHN cohorts as testing sets. The five highest ranked features of both feature sets along with their associated main vibrational mode and molecule are discussed in the following subsections for each classification task. The Raman shift of each feature and of its associated Raman peak, found in the literature, are both reported. Table 2 presents the classification performances for all three classification tasks for both testing sets. The threshold corresponding to the point closest-to-(0,1) corner was selected for each ROC to obtain accuracy, sensitivity, and specificity values reported in Table 2. In all cases, classification performances of the combined model were always equal or higher than results obtained using only one feature type. Furthermore, using the intensity model yielded higher classification performances than using the peak model, except for the classification of cancer/IDC-P tissues for the CHUQc-UL testing set. The intensity model results are superior compared to our previous study (‘previous’ column) for the classification of cancer/IDC-P and HGPIN/IDC-P, whereas the opposite was observed for the benign/cancer classification task.

**Table 2:** Accuracy, sensitivity, specificity, and AUC for the three classification tasks: benign versus cancer, cancer versus IDC-P, and HGPIN versus IDC-P. Classification performances are given for four different models: Results previously published^2^ (previous), intensity (using only individual intensity features, similar as previously published^2^), peak (using only Raman Gaussian fitted peak metrics, i.e., heights and widths), combined (using both intensity and peak features). The combined models always have an equal or better classification performance than the intensity and peak models. The relative change in AUC with respect to our previous study is included in parenthesis in the combined columns.

Performance metric	Classification performance (%±SD)											
	Training				Testing							
	CHUM				UHN CHUQc-UL							
Benign/cancer	Previous	Intensity	Peak	Combined	Previous	Intensity	Peak	Combined	Previous	Intensity	Peak	Combined
Accuracy	87±5	86±2	86±3	85±4	84	83	81	84 (+0)	86	80	79	81 (-5)
Sensitivity	86±6	86±3	87±4	85±6	84	84	81	84 (+0)	87	80	79	80 (-7)
Specificity	89±8	86±3	84±3	85±5	82	80	80	84 **(+2)**	81	78	79	82 (**+1**)
AUC	87±5	90±2	88±2	90±5	83	85	85	87 **(+4)**	84	88	85	90 (+6)
Cancer/IDC-P												
Accuracy	95±2	97±1	95±1	97.0±1	91	94	89	95 **(+4)**	85	88	91	93 **(+8)**
Sensitivity	96±4	97±1	95±1	97.0±1	88	93	89	95 **(+7)**	85	88	91	94 **(+9)**
Specificity	94±2	97±1	94±1	97.0±1.0	93	95	89	95 **(+2)**	86	88	92	92 **(+6)**
AUC	95±3	99.3±0.3	98±1	99.7±0.3	90	97	96	99 **(+9)**	88	93	97	97 **(+9)**
HGPIN/IDC-P												
Accuracy	97.5±1.4	99.5±0.8	95±3	99±2	97.8	100	98	100**(+2.2)**	98.3	100	100	100**(+1.7)**
Sensitivity	98.2±1.5	99.8±0.7	94±5	99±2	95.5	100	96	100 **(+4.5)**	96.4	100	100	100 **(+3.6)**
Specificity	97.1±1.4	99±2	96±4	100±1	100	100	100	100 (+0)	100	100	100	100 (+0)
AUC	97.6±1.4	99.9±0.1	98±2	99.8±0.2	97.7	100	100	100**(+2.3)**	100	100	100	100 (+0)

The method developed in Ref. 19 for the comparison of ROCs was used to test if improvements were statistically significant. A comparison between previous, intensity, and peak model versus the combined model was performed for both testing sets. For each comparison, a p-value was obtained. All model comparisons were statistically significant for the benign/cancer classification (p-value ≤0.05) except for the comparison of intensity and combined models for both testing sets. The p-values were 0.3414 and 0.2696 for the UHN and CHUQc-UL testing set, respectively. While they are not significant, if a 0.05 threshold is adopted, the combination of intensity and peak features is still a non-negligible improvement. All model comparisons were statistically significant for the cancer/IDC-P classification task except for the peak and combined model comparison for the CHUQc-UL testing. The previous and combined model comparison for the UNH testing was the only statistically significant improvement for the HGPIN/IDC-P classification.

### 3.1 Benign/Cancer Classification

The selected hyperparameters, optimized by cross-validated grid-search, for the intensity, peak, and intensity+peak models are : $k_{\text{intensity}}=(15,NA,16)$, $C_{\text{intensity}}^{L1}=(1\times 10^{-4},NA,1\times 10^{-4})$, $n_{\text{neighbor}}=(5,NA,6)$, $k_{\text{peak}}=(NA,6,8)$ with $C_{\text{peak}}^{L1}=(NA,1\times 10^{-6},5\times 10^{-5})$, $C^{\mathrm{RBF}}=(0.07,0.2,0.06)$, and $\gamma^{\mathrm{RBF}}=(5\times 10^{-3},5\times 10^{-4},5\times 10^{-3})$, where NA stands for Not Applicable, e.g., the intensity model does not have a $k_{\text{peak}}$ hyperparameter and is thus NA. Combining the two feature types improved the classification metrics for the UHN and CHUQc-UL testing sets and, in both cases, the intensity model was superior to the peak model. The difference in classification performance [accuracy, sensitivity, specificity, AUC] between the intensity and intensity+peak feature sets for both training sets was: +1%, +0%, +4%, +2. Performance metrics obtained using the combined model compared to our previous study changed according to the following: +0% (−5%), +0% (−7%), +2% (+1%), +4 (+6). While some classification metric decreased, the AUC increased for both testing sets. The results for the training set are similar to the results obtained on the testing sets, i.e., a slight decreased in accuracy (−2%), sensitivity (−1%), specificity (−4%), and an increased in AUC (+3) while retaining similar uncertainties.

**Fig. 2:**
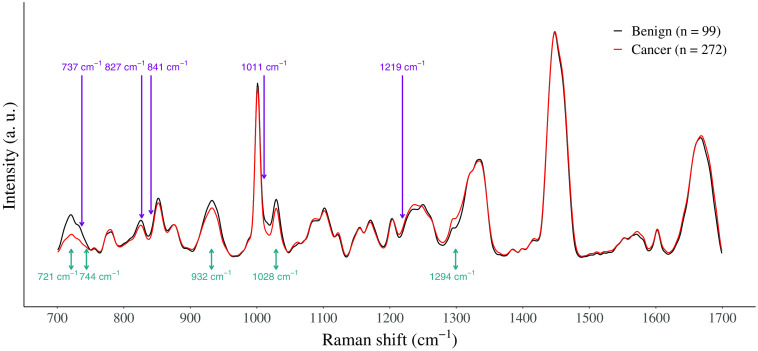
Average Raman spectra of cancer (red, n=272) and benign (black, n=99) tissue annotated with the five highest ranked individual intensity features (purple) and the five highest ranked peak features (teal, for height features).

The average Raman spectra for both benign and cancer prostate tissue and the five highest ranked features of each feature set are shown in Fig. 2. The description of the five best features for each the peak and the intensity models are presented in Table 3 along with their respective molecular assignments.^23^ For the peak height feature set, four Raman peaks ranging from 719 to $1032\;{\mathrm{cm}}^{-1}$ were increased in the average spectrum of benign tissue. DNA, RNA, proteins, and amino acids (i.e., tryptophan, proline, valine, and phenylalanine) were the main biochemical components associated with these peaks. The only feature associated with an increase in cancer tissue was the peak at $1294\;{\mathrm{cm}}^{-1}$, which was assigned to lipids. For the intensity feature set, a unique peak ($1250\;{\mathrm{cm}}^{-1}$) associated with the β-sheet secondary structure of proteins was more intense in the Raman spectrum of cancer tissue. There were also 4 Raman peaks increased in benign tissue, from 719 to $1003\;{\mathrm{cm}}^{-1}$ and their molecular assignments are mostly DNA, RNA, proteins, and amino acids (i.e., tyrosine, proline, and phenylalanine).

**Table 3:** Highest ranked features used for the classification of benign and malignant prostate tissue found using a linear SVM with L1 regularization, and their associated Raman peaks. Tentative molecular assignment of prostate Raman peaks based on the literature. The Raman shift of the features (feature column) and of its associated Raman peak (peak, center column) are reported.^2^^,^^9^^–^^12^^,^^20^^–^^22^

Feature set	Feature position (${\mathrm{cm}}^{-1}$)	Raman peak position (${\mathrm{cm}}^{-1}$)	Tissue type associated with increase	Main vibrational modes	Main molecules
Peak height	721	719–726	Benign	Ring breathing mode, C─S	DNA/RNA (adenine), protein
Peak height	744	742–746	Benign	Ring breathing mode	DNA/RNA (bases, thymine), protein (tryptophan)
Peak height	932	935–937	Benign	C─C stretch	Protein (proline, valine, α-helix)
Peak height	1028	1031–1032	Benign	C─H stretch	Protein (phenylalanine)
Peak height	1294	1296–1305	Cancer	Fatty acid	Lipid
Intensity	737	719–726	Benign	Ring breathing mode, C─S	DNA/RNA backbone, protein (tyrosine)
Intensity	827	827–828	Benign	O─P─O stretch, ring breathing	DNA/RNA backbone, protein (tyrosine)
Intensity	841	853	Benign	C─C stretch, ring breathing	Protein (proline, tyrosine)
Intensity	1011	1000–1003	Benign	Symmetric ring breathing	Protein (phenylalanine)
Intensity	1219	1242–1250	Cancer	Amide III	Protein (β-sheet)

### 3.2 Cancer/IDC-P Classification

The optimal hyperparameters to classify cancer from IDC-P tissue using the (intensity, peak, and intensity+peak) models are: $k_{\text{intensity}}=(77,NA,76)$ with $C_{\text{intensity}}^{L1}=(1\times 10^{-3},NA,5\times 10^{-6})$, $n_{\text{neighbor}}=(7,NA,8)$, $k_{\text{peak}}=(NA,74,90)$ with $C_{\text{peak}}^{L1}=(NA,1\times 10^{-2},1\times 10^{-2})$, $C^{\mathrm{RBF}}=(2,2,5)$, and $\gamma_{\text{peak}}^{\mathrm{RBF}}=(0.01,0.01,0.01)$ where NA stands for Not Applicable. The performance metrics of the combined model for the UHN (CHUQc-UL) testing set improved our previous results by: +4% (+8%), +7% (+9%), +2% (+6%), +9 (+9). It also improved the results on the CHUM training set by: 2%, 1%, 3%, 4.7 while also decreasing the standard deviation by: −1%, −3%, −1%, 2.7.

**Fig. 3:**
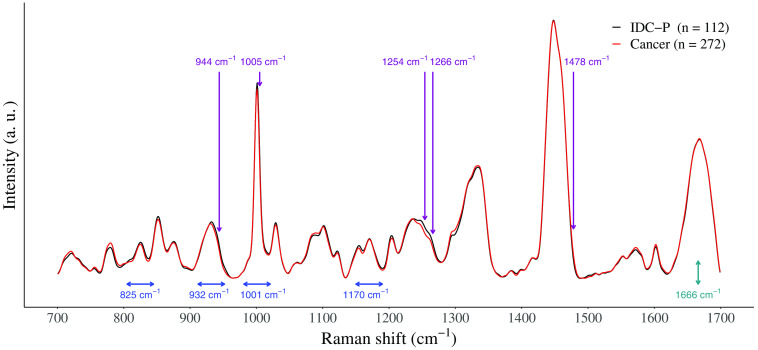
Average Raman spectra of cancer (red, n=272) and IDC-P (black, n=112) tissue along with the 5 highest ranked individual intensity features (purple) and the five highest ranked peak features (teal showing height features; blue showing width features).

The description of the five highest ranked features for each the peak and the intensity models are shown in Fig. 3 and Table 4 along with their respective molecular assignments. Within the five highest ranked peak features, three Raman peaks were increased in the average spectrum of IDC-P compared to cancer tissue. These peaks were mostly associated with amino acids: $825\;{\mathrm{cm}}^{-1}$ (tyrosine), $1170\;{\mathrm{cm}}^{-1}$ (tyrosine), and $932\;{\mathrm{cm}}^{-1}$ (proline and valine). Raman peaks assigned to phenylalanine ($1001\;{\mathrm{cm}}^{-1}$), protein, lipids, DNA, and RNA ($1666\;{\mathrm{cm}}^{-1}$) were reduced in IDC-P. For the intensity feature set, the $1484\;{\mathrm{cm}}^{-1}$ Raman peak, assigned to the nucleobases adenine and guanine, was the single decreased peak in the average spectrum of IDC-P. The four other Raman peaks, ranging from 944 to $1266\;{\mathrm{cm}}^{-1}$, were identified mostly in IDC-P. The biochemical constituents associated with these peaks were predominately linked to proteins: α-helix and β-sheet secondary structures, proline, valine, and phenylalanine.

**Table 4:** Highest ranked features used to classify intraductal carcinoma of the prostate and invasive prostate cancer tissue found using a linear SVM with L1 regularization, and their associated Raman peaks. Tentative molecular assignment of prostate Raman peaks based on the literature. The Raman shift of the features (feature column) and of its associated Raman peak (peak, center column) are reported.^2^^,^^9^^–^^12^^,^^20^^–^^22^

Feature set	Feature position (${\mathrm{cm}}^{-1}$)	Raman peak position (${\mathrm{cm}}^{-1}$)	Tissue type associated with increase	Main vibrational modes	Main molecules
Peak width	825	827–831	IDC-P	O─P─O stretch, ring breathing	DNA/RNA backbone, protein (tyrosine)
Peak height	932	935–937	IDC-P	C─C stretch	Protein (proline, valine, α-helix)
Peak width	1001	1000–1003	Cancer	Symmetric ring breathing	Protein (phenylalanine)
Peak width	1170	1171	IDC-P	C─H bend	Protein (tyrosine)
Peak width	1666	1657–1667	Cancer	C═O stretch, Amide I	Protein (α-helix), lipid (fatty acid), DNA/RNA (thymine)
Intensity	944	935–937	IDC-P	C─C stretch	Protein (proline, valine, α-helix)
Intensity	1005	1000–1003	IDC-P	Symmetric ring breathing	Protein (phenylalanine)
Intensity	1254	1242–1250	IDC-P	Amide III	Protein (β-sheet
Intensity	1266	1263	IDC-P	Amide III	DNA/RNA (thymine, adenine), protein (α-helix)
Intensity	1478	1484	Cancer	Ring breathing mode	DNA/RNA (adenine, guanine)

### 3.3 HGPIN/IDC-P Classification

By using only the individual intensity features a classification with 100% AUC was obtained for both testing sets, and combining both feature sets also yielded the same result. The optimal hyperparameters for all three data sets were $k_{\text{peak}}=42$ with $C_{\text{peak}}^{L1}=1\times 10^{-4}$, $k_{\text{intensity}}=49$ with $C_{\text{intensity}}^{L1}=1\times 10^{-4}$, $C^{\mathrm{RBF}}=3$, $\gamma_{\text{peak}}^{\mathrm{RBF}}=0.003$, and $n_{\text{neighbor}}=4$. The improvements in accuracy, sensitivity, specificity, and AUC with respect to our previous study were: +2.2% (+1.7%), +4.5% (+3.6%), +0% (+0%), +2.3 (+0) for the UHN (CHUQc-UL) testing set. Those results were obtained while also improving the result on the training set (CHUM) by: +2.5%, +0.8%, +2.9%, and +2.4%.

**Fig. 4:**
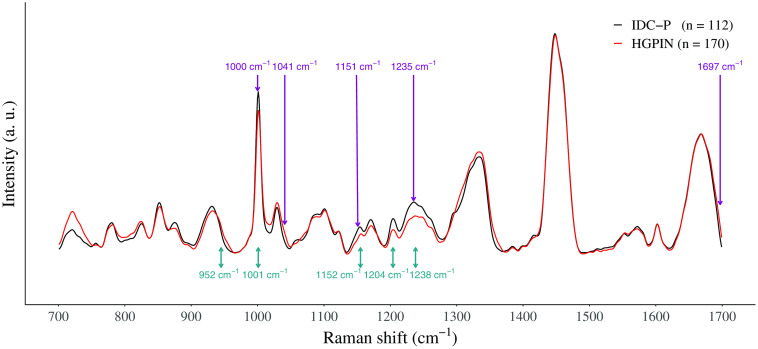
Average Raman spectra of HGPIN (red, n=170) and IDC-P (black, n=112) tissue along with the five highest ranked individual intensity features (purple) and the five highest ranked peak features (teal, for height features).

Following the classification of IDC-P and HGPIN, the five highest ranked features were assigned to specific molecules (Fig. 4 and Table 5). Only one feature was found to be a dominant contributor for identifying HGPIN using the peak height feature set. More specifically, the $937\;{\mathrm{cm}}^{-1}$ Raman peak was identified as α-helix secondary structure of proteins and two amino acids, proline and valine. The other four features, from 1001 to $1238\;{\mathrm{cm}}^{-1}$, were all increased in the average spectrum of IDC-P tissue and were assigned to the β-sheet secondary structure of proteins, phenylalanine, tryptophan, and tyrosine. For the classification using the intensity feature set, two Raman peaks were increased in HGPIN tissue: phenylalanine ($1032\;{\mathrm{cm}}^{-1}$); α-helix secondary structure of proteins, fatty acid, and the nucleobase thymine ($1667\;{\mathrm{cm}}^{-1}$). Raman peaks at $1003\;{\mathrm{cm}}^{-1}$ (phenylalanine) and at $1250\;{\mathrm{cm}}^{-1}$ (β-sheet) were mostly found in IDC-P tissue. The carotenoid biochemical component ($1152\;{\mathrm{cm}}^{-1}$) was a main contributor to the IDC-P classification in both feature sets.

**Table 5:** Highest ranked features used to classify high-grade prostatic intraepithelial neoplasia and IDC-P found using a linear SVM with L1 regularization, and their associated Raman peaks. Tentative molecular assignment of prostate Raman peaks based on the literature. The Raman shift of the features (feature column) and of its associated Raman peak (peak, center column) are reported.^2^^,^^9^^–^^12^^,^^20^^–^^22^

Feature set	Feature position (${\mathrm{cm}}^{-1}$)	Raman peak position (${\mathrm{cm}}^{-1}$)	Tissue type associated with increase	Main vibrational modes	Main molecules
Peak height	952	935–937	HGPIN	C─C stretch	Protein (proline, valine, α-helix)
Peak height	1001	1000–1003	IDC-P	Symmetric ring breathing	Protein (phenylalanine)
Peak height	1152	1152	IDC-P	C─N and C─C stretch	Protein, carotenoid
Peak height	1204	1206–1207	IDC-P	$C-C_{6}H_{5}$ stretch	Protein (phenylalanine, tryptophan, tyrosine)
Peak height	1238	1242–1250	IDC-P	Amide III	Protein (β-sheet)
Intensity	1000	1000–1003	IDC-P	Symmetric ring breathing	Protein (phenylalanine)
Intensity	1041	1031–1032	HGPIN	C─H stretch	Protein (phenylalanine)
Intensity	1151	1152	IDC-P	C─N and C─C stretch	Protein, carotenoid
Intensity	1235	1242–1250	IDC-P	Amide III	Protein (β-sheet)
Intensity	1697	1657–1667	HGPIN	C═O stretch, amide I	Protein (α-helix), lipid (fatty acid), DNA/RNA (thymine)

## 4 Discussion

The key difference in this analysis was the combination of the intensity and peak features, which improves the AUC of all three classification tasks. Combining both types of features had more impact on the classification of IDC-P versus cancer than on benign versus cancer but could not be appropriately quantified for HGPIN versus IDC-P since using only one type of feature was enough to obtain a perfect classification score.

While the accuracy, sensitivity, and specificity of these results are lower than those of our previous study for the classification of benign and malignant prostate tissue, the AUC increased by +4% for the UHN testing set, and +6% for the CHUQc-UL testing set. In comparison, for both testing sets, the AUC increased by 9% for the classification of IDC-P versus cancer. One hypothesis that could explain the higher AUC improvement for the classification of IDC-P versus cancer is the presence of four width features within the five highest ranked features in the IDC-P/cancer classification task and their absence in benign/cancer task (see Tables 3 and 4, respectively). In the results section (Sec. 3) of this analysis, peak centers of individual intensity of Raman bands and heights were reported but also compared to the most important features of our previous study. In this particular case, it is the first time the widths of Raman peaks are identified as being useful for classification purposes in a machine learning setting. Therefore, for now, this observation is only factual and there is no interpretation. Raman spectra datasets on various tissues for various classification tasks are required to learn if the width of peaks are also relevant features for other applications.

Comparing the highest ranked individual intensity features of each classification task with the highest ranked features found in our previous study^2^ reveals that both analyses identified similar features. The intensity features of the cancer/benign and IDC-P/cancer tasks are present within the 10 highest ranked features of our previous analysis and they are also assigned to the type of tissue. As for the HGPIN/IDC-P task, there are only two similar features. However, since in both cases the classification performance is near perfect, the highest ranked features are likely interchangeable due to the large spectral difference between the two tissue types.

The results obtained in this analysis using only the individual intensity features also yielded improved results compared to those obtained in our previous analysis for two classification tasks: cancer/IDP-C and HGPIN/IDC-P.

This is likely the result of a combination of several differences such as using the SVN normalized individual intensities as input for the SVM L1 feature selection algorithm instead of using SNV normalized spectra as in the PLUS study. The addition of the $n_{\text{neighbor}}$ hyperparameter might also have contributed to improving the classification results.

A probable limiting factor for the classification performance of benign/cancer could be the presence of different tumor stages and Gleason scores distributions in the training and testing dataset (see Table 1). For example, the training and testing sets contain 139 and 28 spectra with a Gleason score of ≤3+3, respectively, and contain 22 and 56 spectra with a Gleason score of 4+3 for the training and testing set, respectively. These distribution imbalances, both Gleason score wise and tumor stage wise could explain the lower AUC score for the classification of benign versus cancer tissue in comparison to IDC-P versus cancer. A follow-up study is planned to investigate this.

The most important difference with respect to our previous result is the identification of Raman peak widths as important biomarkers for the classification of IDC-P and invasive prostate cancer tissue. These results further reinforce the usefulness of the clinical implementation of Raman microscopy. By exploring an exhaustive list of feature selection algorithms, models more readily transferable to the clinical workplace with a total number of features of ten or less will be studied.

## Code, Data, and Materials Availability

All Raman spectra files are available from the Dryad Digital Repository database.^16^

## References

[1] Humphrey P. A. et al., “The 2016 who classification of tumours of the urinary system and male genital organs-part b: prostate and bladder tumours,” Europ. Urol. 70, 106–119 (2016). EUURAV 0302-2838 10.1016/j.eururo.2016.02.028 26996659

[2] Grosset A.-A. et al., “Identification of intraductal carcinoma of the prostate on tissue specimens using Raman micro-spectroscopy: a diagnostic accuracy case–control study with multicohort validation,” PLOS Med. 17, e1003281 (2020).1549-1676 10.1371/journal.pmed.1003281 32797086 PMC7428053

[3] Jermyn M. et al., “A review of Raman spectroscopy advances with an emphasis on clinical translation challenges in oncology,” Phys. Med. Biol. 61, R370–R400 (2016). PHMBA7 0031-9155 10.1088/0031-9155/61/23/R370 27804917

[4] Guyon I. et al., “Gene selection for cancer classification using support vector machines,” Mach. Learn. 46, 389–422 (2002). MALEEZ 0885-6125 10.1023/A:1012487302797

[5] Dorigo M., “Optimization, learning and natural algorithms,” PhD., Politecnico diMilano (1992).

[6] Weston J. et al., “Use of the zero norm with linear models and kernel methods,” J. Mach. Learn. Res. 3, 1439–1461 (2003). 10.1162/153244303322753751

[7] Schapire R. E. Singer Y., “Improved boosting algorithms using confidence-rated predictions,” Mach. Learn. 37, 297–336 (1999). MALEEZ 0885-6125 10.1023/A:1007614523901

[8] Kemmler M. Denzler J., “Finding discriminative features for Raman spectroscopy,” in Proc.—Int. Conf. Pattern Recognit.(2012).

[9] Patel I. I. Martin F. L., “Discrimination of zone-specific spectral signatures in normal human prostate using Raman spectroscopy,” Analyst 135, 3060–3069 (2010). ANLYAG 0365-4885 10.1039/c0an00518e 20949203

[r10] Taleb A. et al., “Raman microscopy for the chemometric analysis of tumor cells,” J. Phys. Chem. B 110, 19625–19631 (2006). JPCBFK 1520-6106 10.1021/jp061981q 17004830

[r11] Crow P. et al., “The use of Raman spectroscopy to differentiate between different prostatic adenocarcinoma cell lines,” Br. J. Cancer 92, 2166–2170 (2005). BJCAAI 0007-0920 10.1038/sj.bjc.6602638 15928665 PMC2361812

[r12] Wang L. et al., “Raman spectroscopy, a potential tool in diagnosis and prognosis of castration-resistant prostate cancer,” J. Biomed. Opt. 18(8), 045001 (2013). JBOPFO 1083-3668 10.1117/1.JBO.18.4.045001 23907278

[r13] Tollefson M. et al., “Raman spectral imaging of prostate cancer: can Raman molecular imaging be used to augment standard histopathology?” BJU Int. 106(4), 484–488 (2010). BJINFO 1464-410X 10.1111/j.1464-410X.2010.09185.x 20201840

[14] Crow P. et al., “The use of Raman spectroscopy to identify and grade prostatic adenocarcinoma in vitro,” Br. J. Cancer 89, 106–108 (2003). BJCAAI 0007-0920 10.1038/sj.bjc.6601059 12838309 PMC2394218

[15] Lemoine E. et al., “Feature engineering applied to intraoperative in vivo Raman spectroscopy sheds light on molecular processes in brain cancer: a retrospective study of 65 patients,” Analyst 144, 6517–6532 (2019). ANLYAG 0365-4885 10.1039/C9AN01144G 31647061

[16] Grosset A.-A. et al., “Data from: Identification of intraductal carcinoma of the prostate on tissue specimens using Raman micro-spectroscopy: a diagnostic accuracy case-control study with multicohort validation,” Dryad Dataset, https://datadryad.org/stash/dataset/doi:10.5061/dryad.cjsxksn3p. 10.1371/journal.pmed.1003281 PMC7428053 32797086

[17] Perez-Pueyo R. Soneira M. J. Ruiz-Moreno S., “Morphology-based automated baseline removal for Raman spectra of artistic pigments,” Appl. Spectrosc. 64(6), 595–600 (2010). APSPA4 0003-7028 10.1366/000370210791414281 20537226

[18] R Core Team, “R: a language and environment for statistical computing,” R Foundation for Statistical Computing, Vienna, Austria (2021).

[19] DeLong E. R. DeLong D. M. Clarke-Pearson D. L., “Comparing the areas under two or more correlated receiver operating characteristic curves: a nonparametric approach,” Biometrics 44(3), 837–845 (1988). BIOMB6 0006-341X 10.2307/2531595 3203132

[20] Devpura S. et al., “Detection of benign epithelia, prostatic intraepithelial neoplasia, and cancer regions in radical prostatectomy tissues using Raman spectroscopy,” Vib. Spectrosc. 53(2), 227–232 (2010). VISPEK 0924-2031 10.1016/j.vibspec.2010.03.009

[r21] Kast R. et al., “Emerging technology: applications of Raman spectroscopy for prostate cancer,” Cancer Metastasis Rev. 33 (2014). 10.1007/s10555-013-9489-6 24510129

[22] Talari A. C. S. et al., “Raman spectroscopy of biological tissues,” Appl. Spectrosc. Rev. 50(1), 46–111 (2015). APSRBB 0570-4928 10.1080/05704928.2014.923902

[23] Gandhi J. et al., “Reporting practices and resource utilization in the era of intraductal carcinoma of the prostate: a survey of genitourinary subspecialists,” Am. J. Surg. Pathol. 44, 673–680 (2019). 10.1097/PAS.0000000000001417 31876580

